# Activity of a Long-Acting Injectable Bedaquiline Formulation in a Paucibacillary Mouse Model of Latent Tuberculosis Infection

**DOI:** 10.1128/AAC.00007-19

**Published:** 2019-03-27

**Authors:** Amit Kaushik, Nicole C. Ammerman, Sandeep Tyagi, Vikram Saini, Iwan Vervoort, Sophie Lachau-Durand, Eric Nuermberger, Koen Andries

**Affiliations:** a Center for Tuberculosis Research, Department of Medicine, Johns Hopkins University School of Medicine, Baltimore, Maryland, USA; b Janssen R&D, Beerse, Belgium

**Keywords:** BALB/c mice, bedaquiline, latent tuberculosis infection, long-acting injectable

## Abstract

The potent antituberculosis activity and long half-life of bedaquiline make it an attractive candidate for use in long-acting/extended-release formulations for the treatment of latent tuberculosis infection (LTBI). Our objective was to evaluate a long-acting injectable (LAI) bedaquiline formulation in a validated paucibacillary mouse model of LTBI. Following immunization with Mycobacterium bovis rBCG30, BALB/c mice were challenged by aerosol infection with M. tuberculosis H37Rv. Treatment began 13 weeks after challenge infection with one of the following regimens: an untreated negative-control regimen; positive-control regimens of daily rifampin (10 mg/kg of body weight), once-weekly rifapentine (15 mg/kg) and isoniazid (50 mg/kg), or daily bedaquiline (25 mg/kg); test regimens of one, two, or three monthly doses of LAI bedaquiline at 160 mg/dose (B_LAI-160_); and test regimens of daily bedaquiline at 2.67 mg/kg (B_2.67_), 5.33 mg/kg (B_5.33_), or 8 mg/kg (B_8_) to deliver the same total amount of bedaquiline as one, two, or three doses of B_LAI-160_, respectively. All drugs were administered orally, except for B_LAI-160_ (intramuscular injection). The primary outcome was the decline in M. tuberculosis lung CFU counts during 12 weeks of treatment. The negative- and positive-control regimens performed as expected. One, two, and three doses of B_LAI-160_ resulted in decreases of 2.9, 3.2, and 3.5 log_10_ CFU/lung, respectively, by week 12. Daily oral dosing with B_2.67_, B_5.33_, and B_8_ decreased lung CFU counts by 1.6, 2.8, and 4.1 log_10_, respectively. One dose of B_LAI-160_ exhibited activity for at least 12 weeks. The sustained activity of B_LAI-160_ indicates that it shows promise as a short-course LTBI treatment requiring few patient encounters to ensure treatment completion.

## Abstract (precis)

The potent antituberculosis activity and long half-life of bedaquiline make it an attractive candidate for use in long-acting/extended-release formulations for the treatment of latent tuberculosis infection (LTBI). Our objective was to evaluate a long-acting injectable (LAI) bedaquiline formulation in a validated paucibacillary mouse model of LTBI.

## INTRODUCTION

The crux of global efforts to eliminate tuberculosis (TB) is the prevention of Mycobacterium tuberculosis transmission in the population. With approximately 10 million incident cases of TB occurring annually (1), the identification and treatment of individuals with active disease are clearly necessary for interrupting transmission. Addressing incident TB in the population, however, will not be sufficient to end the global epidemic, as the World Health Organization (WHO) has stated that up to one-third of the world’s population may be latently infected with M. tuberculosis, referred to as latent TB infection (LTBI) (2). This reservoir of potentially billions of people serves as an ever-present source of new TB cases, independent of recent transmission events (3). Thus, the identification and treatment of individuals with LTBI are also essential components of WHO’s End TB strategy (2, 4).

There are currently four WHO-recommended LTBI treatment regimens: daily isoniazid monotherapy for 6 to 9 months, daily rifampin for 3 to 4 months, daily rifampin plus isoniazid for 3 months, and weekly rifapentine plus isoniazid for 3 months (2). When completed, all four regimens are highly efficacious in reducing the risk of developing active TB disease, but the shorter (i.e., 3- to 4-month) regimens are associated with higher rates of completion than the longer (i.e., 6- to 9-month) regimen (5–7). However, ensuring treatment completion of the 3- or 4-month regimens is still a formidable challenge for TB control programs. The availability of efficacious regimens even shorter than 3 months could further improve treatment completion rates. An example is the 1-month daily rifapentine-plus-isoniazid regimen recently evaluated in a phase 3 clinical trial for the treatment of LTBI in individuals infected with HIV (8); the efficacy of this regimen was not inferior to that of the 9-month control regimen of daily isoniazid and was associated with statistically significantly higher rates of treatment completion.

Beyond decreasing regimen durations, another modification with the potential to significantly reduce the burden on health care delivery systems and improve LTBI treatment completion rates is the use of long-acting injectable (LAI) formulations for drug administration (9). The development and administration of LAI and implantable drug formulations have improved adherence, i.e., there are fewer missed daily doses following injection, compared to that with daily oral drug intake in patients receiving antipsychotic medications (10) and in individuals using hormone-based contraceptives (11). Recently, significant advances have been made in the development of LAI formulations of antiretroviral drugs administered monthly or bimonthly for both the prevention and treatment of HIV infection (12–15), with LAI formulations of cabotegravir and rilpivirine currently being evaluated in a phase 3 randomized clinical trial in adults with HIV-1 infection (ClinicalTrials.gov identifier NCT02951052). LAI formulations may be easier for children than formulations that require them to swallow daily medications (16) (ClinicalTrials.gov identifier NCT03497676), and importantly, several studies have indicated a high level of interest in and preference for long-acting injectable forms versus daily oral administration of HIV preexposure prophylaxis across diverse populations (17–20).

Although LAI formulations may be well suited for use in LTBI treatment, not all anti-TB drugs are well suited for use in LAI formulations. Two key properties of (pro)drugs administered in LAI formulations are low aqueous solubility, to preclude the rapid dissolution and release of the active drug substance, and a reasonably long pharmacokinetic (PK) elimination half-life, i.e., slow clearance from the body (21). For an antimicrobial, another desired property is high potency, negating the need for high concentrations in the blood (9) and allowing low drug doses to be injected. The diarylquinoline bedaquiline, which has a low MIC for M. tuberculosis (about 0.03 μg/ml), high lipophilicity (logP, 7.3), and a long half-life (about 24 h, functionally or effectively), possesses a profile that may be suitable for use in an LAI formulation (9, 22–25). Furthermore, bedaquiline has been shown to specifically contribute significant treatment-shortening activity in mouse models of TB (26–28) and is associated with treatment shortening in patients with multidrug-resistant (MDR) TB (29–31), suggesting that this drug could also contribute treatment-shortening activity to an LTBI treatment regimen. We previously demonstrated in a validated paucibacillary mouse model of LTBI that 3 months of daily, orally administered bedaquiline had sterilizing activity equivalent or superior to that of each of the four WHO-recommended regimens (32). Thus, an LAI formulation of bedaquiline has the potential to significantly shorten and simplify LTBI treatment, including MDR-LTBI treatment.

Here, we describe the PK and activity of an LAI bedaquiline formulation in the validated paucibacillary mouse model of LTBI (32–34) by comparing the bactericidal activity of one, two, or three monthly injections of the long-acting formulation to that of the same total doses of bedaquiline administered daily by the oral route.

## RESULTS

### MIC of the long-acting bedaquiline formulation for M. tuberculosis H37Rv.

Using a broth macrodilution assay, the MIC of the long-acting bedaquiline microsuspension formulation for M. tuberculosis H37Rv was 0.03 μg/ml. This was identical to the MIC of the bedaquiline powder used for oral drug administration and in agreement with previously published MIC values of bedaquiline for this strain (28, 35).

### PK characteristics of long-acting bedaquiline formulation.

**FIG 1:**
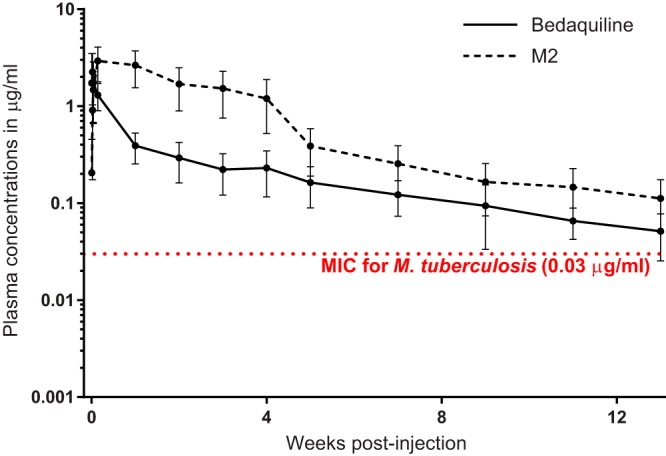
Plasma concentrations of bedaquiline and its M2 metabolite following a single, 160-mg/kg intramuscular injection of long-acting bedaquiline. Data points represent mean values, and error bars represent standard deviations (5 mice sampled per time point).

The mouse plasma concentration-time profiles of bedaquiline and its M2 metabolite are displayed in Fig. 1, and the total drug plasma PK parameters are presented in Table 1. After intramuscular administration of 160 mg/kg of body weight of the bedaquiline microsuspension, the release of bedaquiline from the injection site was slow, as shown by plasma concentrations above the MIC at 2,184 h (13 weeks) postdosing. The peak plasma concentration (*C*_max_) of bedaquiline was reached earlier (range, 1 to 4 h) than that of M2 (range, 24 to 168 h). Considering the interindividual variability, the *C*_max_ values were not significantly different between bedaquiline and M2, whereas the value of the area under the concentration-time curve from time zero to infinity (AUC_0–∞_) was 3.6-fold higher for M2 than for bedaquiline.

**TABLE 1:** Plasma pharmacokinetic parameters of bedaquiline and M2 after a single, 160-mg/kg intramuscular injection of long-acting bedaquiline^*a*^

Pharmacokinetic parameter	Values for:	
	Bedaquiline	M2
*C*_max_ (ng/ml)	2,363 (1,447)	3,002 (1,139)
*T*_max_ (h)^*a*^	1–4	24–168
AUC_0–2,184_ (ng·h/ml)	447,361 (174,979)	1,689,422 (755,169)
AUC_0–∞_ (ng·h/ml)	500,850 (201,928)	1,823,672 (836,740)

a A range is given for *T*_max_ values; all other data represent the means (standard deviations) for five mice sampled at each time point.

### Establishment of stable, low-level M. tuberculosis infection.

Mice were immunized by aerosol infection with M. bovis rBCG30; on the day after immunization, the mean rBCG30 lung CFU count was 3.05 log_10_ CFU/lung (standard deviation [SD], 0.10 log_10_ CFU/lung) (see Tables S1 and S2 in the supplemental material). Six weeks later, mice were challenged by aerosol infection with M. tuberculosis H37Rv. On the day after challenge infection, the mean (SD) M. tuberculosis lung CFU count was 2.11 (0.09) log_10_ CFU/lung and the mean (SD) rBCG30 lung CFU count was 4.95 (0.11) log_10_ CFU/lung (Tables S1 and S2). Thirteen weeks later, on the day of treatment initiation (day 0), the mean (SD) M. tuberculosis lung CFU count was 4.75 (0.27) log_10_ CFU/lung, and the mean (SD) lung CFU counts for the untreated control mice (Table 2) remained at this level throughout the experiment: 4.71 (0.48), 4.60 (0.27), and 4.94 (0.29) log_10_ CFU/lung at weeks 4, 8, and 12, respectively (Table 3, Fig. S1, and Tables S3 to S6). Thus, a stable, relatively low-level M. tuberculosis lung infection was established in the mice.

**TABLE 2:** Regimens evaluated for 12 weeks in a paucibacillary mouse model of LTBI treatment

Regimen name	Regimen description
Untreated	Negative control, no drug administered
R_10_ (5/7)	Positive control, rifampin (R) at 10 mg/kg administered daily^*a*^ by gavage
H_50_P_15_ (1/7)	Positive control, isoniazid (H) at 50 mg/kg and rifapentine (P) at 15 mg/kg administered once weekly by gavage^*b*^
B_25_ (5/7)	Positive control, bedaquiline at 25 mg/kg administered daily by gavage
B_8_ (5/7)	Bedaquiline at 8 mg/kg administered daily by gavage
B_5.33_ (5/7)	Bedaquiline at 5.33 mg/kg administered daily by gavage
B_2.67_ (5/7)	Bedaquiline at 2.67 mg/kg administered daily by gavage
B_LAI-160_ (1/28) × 3	Long-acting injectable bedaquiline formulation (B_LAI_) at 160 mg/kg administered every 28 days by intramuscular injection for three total doses: day 0, day 28 (wk 4), and day 56 (wk 8)
B_LAI-160_ (1/28) × 2	B_LAI_ at 160 mg/kg administered every 28 days by intramuscular injection for two total doses: day 0 and day 28 (wk 4)
B_LAI-160_ (1/28) × 1	B_LAI_ at 160 mg/kg administered once on day 0

a Daily indicates administration Monday through Friday.

b Isoniazid was administered at least 1 h after rifapentine administration. The once weekly gavage occurred every Monday.

**TABLE 3:** M. tuberculosis lung CFU counts^*a*^

Regimen	Total bedaquiline dose (mg/kg) administered in 12 wk	Mean (SD) no. of M. tuberculosis log_10_ CFU/lung at the following time points:				
		Wk −13	Day 0	Wk 4	Wk 8	Wk 12
Untreated	NA	2.11 (0.09)	4.75 (0.27)	4.71 (0.48)	4.60 (0.27)	4.94 (0.29)
R_10_ (5/7)	NA			3.39 (0.46)	2.74 (0.62)	1.27 (0.85)
H_50_P_15_ (1/7)	NA			2.67 (0.25)	0.79 (0.80)	0.28 (0.41)
B_25_ (5/7)	1,500			3.01 (0.45)	0.82 (0.49)	0.07 (0.09)
B_8_ (5/7)	480			3.30 (0.12)	2.42 (0.26)	0.69 (0.43)
B_5.33_ (5/7)	320			3.83 (0.25)	3.15 (0.47)	1.98 (0.17)
B_2.67_ (5/7)	160			3.96 (0.35)	3.52 (0.38)	3.16 (0.24)
B_LAI-160_ (1/28) × 3	480					1.23 (0.16)
B_LAI-160_ (1/28) × 2	320				2.31 (0.40)	1.63 (0.40)
B_LAI-160_ (1/28) × 1	160			3.55 (0.32)	3.31 (0.38)	1.83 (0.34)

a Week −13 is the day after aerosol infection with M. tuberculosis; day 0 is the day of treatment initiation. Data are presented graphically in Fig. S1 in the supplemental material. See Table 2 for a description of the regimens. See Tables S2 to S7 for the raw CFU data at each time point. NA, not applicable.

### Activity of LAI bedaquiline in a mouse model of LTBI.

On day 0, treatment was initiated with the regimens described in Table 2. Throughout the 12 weeks of treatment, the daily rifampin and the once-weekly rifapentine-isoniazid control regimens performed as expected (32–34), resulting in total reductions of about 3.5 and 4.5 log_10_ CFU/lung, respectively (Table 3, Fig. S1A, and Tables S3 to S7). Daily oral dosing with bedaquiline at 25 mg/kg also performed as expected (32, 33), resulting in a reduction of about 4.7 log_10_ CFU/lung over 12 weeks of treatment.

For oral bedaquiline regimens, increasing bactericidal activity was observed with increasing dose at weeks 4, 8, and 12 (Table 3, Fig. S1B, and Tables S3 to S7). After 12 weeks of treatment, only the regimen of daily bedaquiline at 2.67 mg/kg (B_2.67_) (5/7) (where 5/7 indicates 5 daily treatments every 7 days) was significantly less bactericidal than the control regimen of rifampin administered at 10 mg/kg daily (R_10_) (5/7) (*P* < 0.0001). For mice that received one or two injections of LAI bedaquiline at 160 mg/dose (B_LAI-160_) (1/28) (where 1/28 indicates one treatment every 28 days), lung CFU counts were equivalent to those in mice that received the same total bedaquiline dose administered as a daily oral regimen at 8 mg/kg (B_8_) (5/7) for 4 or 8 weeks, respectively (*P* > 0.05). The lung CFU counts in mice that received a single injection of B_LAI-160_ declined between each time point (Fig. S1C). After 12 weeks of treatment, their CFU counts were lower than those observed in mice that received the same total dose of bedaquiline (160 mg/kg) via daily oral dosing with B_2.67_ (5/7) (*P* = 0.0002), with the former regimen resulting in a decline of about 3 log_10_ CFU/lung and the latter resulting in a decline of 1.7 log_10_ CFU/lung compared to the count in untreated control mice (Fig. S1D). In mice that received a total bedaquiline dose of 320 mg/kg either through two injections of B_LAI-160_ or through daily oral dosing of daily bedaquiline at 5.33 mg/kg (B_5.33_) (5/7), the decline in lung CFU counts was similar at about 3 log_10_ CFU/lung (*P* > 0.05) (Fig. S1E). For mice that received a total bedaquiline dose of 480 mg/kg via three injections of B_LAI-160_ (1/28), the lung CFU counts were modestly higher than those in mice that received the equivalent total dose through daily oral dosing with B_8_ (5/7) (Fig. S1F), and the difference was not statistically significant.

## DISCUSSION

Although highly efficacious regimens exist for the treatment of LTBI, low treatment completion rates hinder their practical effectiveness (2, 6, 7, 36). The use of LAI drug formulations for LTBI treatment could simplify treatment and improve completion rates (9). It may also overcome limitations of poor or variable oral bioavailability, mitigate concentration-dependent toxicity and drug-drug interactions, and ease administration in children. A bedaquiline LAI-based LTBI regimen could confer these benefits to contacts of MDR-TB patients, for whom short-course rifamycin-containing regimens are likely to be ineffective as preventive therapy. Here, we report the development and initial evaluation of an LAI formulation of bedaquiline with a promising PK and pharmacodynamic (PK) profile for use in LTBI treatment.

While the bacterial burden associated with human LTBI is unknown, it is considered to be at least less than 4 log_10_ CFU since the lower limit of detection by acid-fast smear (the least sensitive method for detecting mycobacteria in sputum) is approximately 4 log_10_ CFU/ml (37–39), and latent lesions found at autopsy can be culture positive but smear negative (40, 41). Although mice are often not considered to develop latent infection with M. tuberculosis, the model used in this study produces a stable, chronic paucibacillary lung infection with a burden that is ≤4 log_10_ CFU/lung (32–34, 42). Thus, this model can be used to evaluate the activity of drugs and regimens against an overall stable M. tuberculosis population present at a burden representing the presumed upper limit of infection in human LTBI. Moreover, this model has been validated through a number of experiments demonstrating the equivalent efficacy of the three rifamycin-based LTBI regimens currently recommended by the WHO (32–34, 43), as well as the 1-month daily isoniazid-rifapentine regimen recently evaluated in a phase 3 clinical trial (8). Furthermore, the superiority of these rifamycin-based regimens to 6 months of isoniazid in this model is consistent with the results of a meta-analysis of clinical trials suggesting that rifamycin-based regimens may have superior efficacy (44). Thus, experimental and clinical evidence supports the use of this model for the preclinical evaluation of LTBI treatment regimens.

In the present experiment, our M. tuberculosis challenge infection resulted in an infectious dose larger than expected. Our goal was to implant approximately 1.0 to 1.5 log_10_ CFU/lung (32–34), but our actual implantation was slightly more than 2 log_10_ CFU/lung. Our previous work has demonstrated that a stable bacterial burden at about 4 log_10_ CFU/lung is achievable only if the implantation is 1.0 to 1.5 log_10_ CFU (i.e., about 30 CFU per mouse lung). (34, 42) As a result, the M. tuberculosis lung burden in this study was somewhat higher than intended at the start of treatment (day 0), averaging 4.75 log_10_ CFU (Table 3). Mice were immunized with rBCG30, a recombinant strain in the Tice BCG background that overexpresses the M. tuberculosis 30-kilodalton major secretory protein (45). This strain is more immunogenic in humans and in mice than the Tice BCG parent strain (34, 46). Interestingly, when BALB/c mice were immunized with the parent BCG strain (and M. tuberculosis implantation was 1.0 to 1.5 log_10_ CFU/lung), the M. tuberculosis lung burden plateaued at about 5 log_10_ CFU/lung (34), which is similar to the burden obtained in our study when the implantation was higher than expected. This bacterial burden is still significantly lower than what would be expected in nonimmunized mice, in which lung CFU counts plateau at about 6 to 7 log_10_ CFU/lung (34, 47). In this study, the M. tuberculosis lung burden remained stable in untreated control mice throughout the duration of the experiment, indicating that the model was still suitable for the evaluation of LTBI regimens. Indeed, the bactericidal activity of the control regimens R_10_ (5/7), isoniazid at 50 mg/kg and rifapentine at 15 mg/kg (H_50_P_15_) (1/7) (where 1/7 indicates that the regimen was administered once a week), and bedaquiline at 25 mg/kg (B_25_) (5/7), which resulted in decreases of 3, 4.5, and 4.9 log_10_ CFU/lung, respectively, after 12 weeks of treatment, was of the same magnitude as that observed in previous studies (32–34). Thus, the higher implantation and day 0 CFU counts did not affect the relative activity of the drugs against this stable bacterial population in the mouse lungs. In addition, in the present experiment, treatment was initiated 13 weeks after M. tuberculosis challenge infection. In this model, treatment is usually started 6 weeks after the challenge infection; however, the start of treatment in this experiment was postponed by an additional 7 weeks due to a delay in the availability of the B_LAI-160_ formulation. This longer incubation period before treatment initiation did not negatively impact the performance of the model. The burden of rBCG30 remained stable in the lungs throughout the treatment period; the mean (SD) count of rBCG30 in the lungs of untreated mice was 3.27 (0.45), 3.27 (0.58), 3.47 (0.53), and 3.07 (0.49) log_10_ CFU/lung at day 0 and weeks 4, 8, and 12, respectively (Tables S3, S4, S5, and S7). As stated above, the burden of M. tuberculosis also remained stable in the lungs of the untreated mice over the course of the experiment. Therefore, there was no apparent decrease in rBCG30-induced immunity in the mice associated with the delay in treatment initiation.

One of the most striking findings from this study was the apparent duration of bactericidal activity associated with a single dose of the long-acting bedaquiline formulation. One injection of B_LAI-160_ at day 0 continued to exert bactericidal activity up to the 12-week time point; these data are supported by the PK data indicating that the plasma bedaquiline levels remained above the MIC for M. tuberculosis for at least 12 weeks postadministration. It is also interesting that, in this experiment, the single injection of B_LAI-160_ exhibited significantly greater bactericidal activity at 12 weeks than the B_2.67_ daily oral regimen that delivered the same total drug dose (160 mg/kg) over 12 weeks. As the mice receiving the other B_LAI-160_ regimens (and the corresponding daily oral dosing regimens) were not followed for a similar amount of time following the last injection of B_LAI-160_, it is not yet possible to know if this apparent increased activity of B_LAI-160_ is reproducible. Additional studies with a longer follow-up after dosing are clearly needed to understand the full extent of the activity of the LAI formulation. When considering this prolonged bactericidal activity, it is possible that just two injections of the long-acting bedaquiline formulation spaced 4 weeks apart could be as active as any of the WHO-recommended LTBI treatment regimens. This is a timely finding, as the recently reported success of the 1-month daily isoniazid-rifapentine regimen (8) may have set a new bar for a short-course LTBI regimen. Even more promising is the idea of combining a single injection of the long-acting bedaquiline formulation with a compatible 1- or 2-week oral regimen. Such a further decrease in patient-provider encounters could revolutionize LTBI treatment. In addition, our results suggest that an LAI formulation of bedaquiline could meet most, if not all, criteria in a recently proposed target product profile for LTBI treatment using LAI formulations, including many of the criteria for an ideal regimen (9). Finally, currently recommended short-course regimens for LTBI, as well as the newly reported 1-month isoniazid-rifapentine regimen, all contain isoniazid and/or a rifamycin (2, 8) and are therefore not expected to be effective against LTBI caused by MDR M. tuberculosis. The present results further support bedaquiline-based regimens for LTBI treatment in contacts of patients with MDR-TB (48), and LAI formulations in particular could significantly simplify what could be an otherwise long and complicated treatment.

## MATERIALS AND METHODS

### Long-acting bedaquiline formulation.

A long-acting formulation of bedaquiline was developed as a microsuspension containing bedaquiline and *d*-tocopherol polyethylene glycol 1000 succinate in a ratio of 4:1 and 50 mg/ml of mannitol. The concentration of bedaquiline in the final formulation was 200 mg/ml.

### PK studies.

The mouse PK study procedures were approved by the local Johnson & Johnson Ethical Committee. Male Swiss mice (4 to 5 weeks old) were purchased from the Janvier Breeding Center (Le Genest Saint-Isle, France). All animals were housed under controlled conditions (specific pathogen free, 23°C, 60% humidity, and a normal light-dark cycle) and had access to food and water *ad libitum*. A single, 160-mg/kg dose of long-acting bedaquiline was administered by intramuscular injection to five mice. Blood samples were taken from each animal at 1, 4, 7, 24, 168, 336, 504, 672, 840, 1,176, 1,512, 1,848, and 2,184 h after injection. Within 1 h of sampling, the blood samples were centrifuged. After centrifugation, plasma was collected and stored at −18°C. At all times, blood/plasma samples were protected from light and placed on melting ice. To measure the levels of bedaquiline and its M2 metabolite in plasma, all samples were analyzed using a qualified liquid chromatograph-tandem mass spectrometry (LC-MS/MS) method (49). The samples were subjected to a selective sample cleanup, followed by LC-MS/MS. The concentrations in the samples were quantified against calibration curves prepared to cover the concentration range of the study samples. The samples used to establish the curves were prepared in the same matrix as the study samples. For each analytical batch, independent quality control samples, prepared in the same matrix as the samples, were analyzed together with the study samples and calibration curve. Individual plasma concentration-time profiles were subjected to a noncompartmental analysis using the linear up/log down trapezoidal rule for all data. The peak plasma concentrations (*C*_max_), the corresponding times to *C*_max_ (*T*_max_), and the area under the plasma concentration-time curve from time zero to time *t* (AUC_0–_*_t_*; where *t* is the sampling time corresponding to the last measurable concentration above the limit of quantification [5 ng/ml]), and from time zero to infinity (AUC_0–∞_) were calculated. All PK analyses were based on total drug concentrations, not adjusted for protein binding, as this has been shown to be a more relevant measure for PK/PD studies of bedaquiline (49).

### Mycobacterial strains.

M. bovis rBCG30, a recombinant strain in the Tice BCG background that overexpresses the M. tuberculosis 30-kilodalton major secretory protein (45), originally provided by Marcus A. Horwitz, and M. tuberculosis H37Rv, American Type Culture Collection strain ATCC 27294, were separately mouse passaged and frozen in aliquots. Frozen stocks were thawed and grown in liquid culture medium to an optical density at 600 nm of about 1.0, and the actively growing cultures were diluted for use in the experiments, as follows: 10-fold in assay medium to prepare the MIC assay inoculum (H37Rv only) and 50-fold (rBCG30) or 100-fold (H37Rv) in phosphate-buffered saline to prepare the suspensions used for infections.

### Media.

The liquid culture medium was Middlebrook 7H9 broth supplemented with 10% (vol/vol) oleic acid-albumin-dextrose-catalase (OADC) enrichment, 0.5% (vol/vol) glycerol, and 0.1% (vol/vol) Tween 80. Assay medium for MIC determination was 7H9 broth supplemented with 10% (vol/vol) OADC and 0.5% (vol/vol) glycerol but without Tween 80. All plating was done on 7H11 agar supplemented with 10% (vol/vol) OADC enrichment and 0.5% (vol/vol) glycerol. Lung homogenates (and their cognate 10-fold dilutions) were plated on selective 7H11 agar (7H11 agar containing 50 μg/ml carbenicillin, 10 μg/ml polymyxin B, 20 μg/ml trimethoprim, and 50 μg/ml cycloheximide) (50) that was further supplemented with 0.4% activated charcoal to adsorb any drug carried over in the homogenates (28). For differentiating M. bovis rBCG30 from M. tuberculosis, selective 7H11 agar was additionally supplemented with either 40 μg/ml hygromycin B, selective for M. bovis rBCG30 and not M. tuberculosis, or 2-thiophenecarboxylic acid hydrazide (TCH), selective for M. tuberculosis and not M. bovis, at 4 and 200 μg/ml in non-charcoal-containing and charcoal-containing agar, respectively. Difco Middlebrook 7H9 broth powder, Difco Mycobacteria 7H11 agar powder, and BBL Middlebrook OADC enrichment were obtained from Becton, Dickinson and Company. Glycerol and Tween 80 were obtained from Fisher Scientific, and activated charcoal was obtained from J. T. Baker. All selective drugs were obtained from Sigma-Aldrich/Millipore-Sigma.

### MIC assays.

The MICs of rifampin, isoniazid, rifapentine, and bedaquiline for our M. tuberculosis H37Rv stock strain were previously determined using the broth macrodilution method and are 0.25, 0.03, 0.03 to 0.06, and 0.06 μg/ml, respectively (28, 35, 51). The same broth macrodilution method was used to compare the MIC of the long-acting bedaquiline formulation with that of orally administered bedaquiline; all forms of bedaquiline were provided by Janssen. M. tuberculosis H37Rv was inoculated into polystyrene tubes containing 2.5 ml assay broth (at 5 log_10_ CFU/ml) containing bedaquiline concentrations ranging (by 2-fold serial dilutions) from 64 to 0.0039 μg/ml. The MIC was defined as the lowest concentration that inhibited visible bacterial growth after 14 days of incubation at 37°C.

### LTBI mouse model.

The mouse model procedures were approved by the Johns Hopkins University Animal Care and Use Committee. The paucibacillary mouse model used in this study was previously described (32–34). Female BALB/c mice (*n* = 150) aged 10 weeks were purchased from Charles River Laboratories. Mice were housed in individually ventilated cages (up to five mice per cage) with sterile wood shavings for bedding and had access to food and water *ad libitum*. Room temperature was maintained at 22 to 24°C, and a 12-h light/12-h dark cycle was used. At each time point, mice were sacrificed by intentional isoflurane overdose by inhalation (drop method), followed by cervical dislocation. Mice were immunized by aerosol infection with a nebulized suspension of M. bovis rBCG30 using a Glas-Col full-size inhalation exposure system per the manufacturer’s instruction; on the day after infection, five mice were sacrificed to determine M. bovis rBCG30 lung implantation. At 6 weeks after the immunizing infection, mice were challenged by aerosol infection with a nebulized suspension of M. tuberculosis H37Rv, and on the day after infection, five mice were sacrificed to determine both the M. tuberculosis lung implantation and the level of M. bovis rBCG30 lung infection. The bacterial concentrations of the suspensions used for infections and the subsequent lung implantation CFU counts were determined as previously described (34, 52) and as outlined in Tables S1 and S2 in the supplemental material, respectively.

### Treatment.

Treatment was initiated on day 0, 13 weeks after M. tuberculosis challenge infection. This was about twice as long as the usual incubation period but was necessitated by a delay in the availability of the LAI formulation. Five mice were sacrificed on day 0 to determine pretreatment bacterial lung levels as previously described (34) and as outlined in Table S3. Mice were randomized into 1 of the 10 treatment regimens described in Table 2. The doses of rifampin, isoniazid, and rifapentine were chosen to achieve plasma exposures (based on the area under the plasma concentration-time curve) in mice similar to those achieved with recommended human doses for the treatment of LTBI (53, 54). The daily bedaquiline dose of 25 mg/kg represents the standard dose used in TB treatment studies in mice (22, 28). The daily oral bedaquiline doses of 2.67, 5.33, and 8 mg/kg were chosen to administer the same total amount of bedaquiline over a 12-week period as one, two, and three doses, respectively, of the LAI bedaquiline formulation, which was administered at 160 mg/kg per dose. For drugs administered by gavage (all drugs except for the long-acting bedaquiline formulation), drug solutions were prepared to deliver the desired dose based on an average mouse body mass of 20 g in a volume of 0.2 ml; drug solutions were prepared weekly and stored at 4°C. Rifampin and isoniazid were purchased from Millipore-Sigma and prepared in distilled water; rifapentine (Priftin) tablets were purchased from a local pharmacy and prepared in distilled water. Orally administered bedaquiline was dissolved in 20% (wt/vol) 2-hydroxypropyl-β-cyclodextrin adjusted to a pH of ∼2 with 1 N HCl. The LAI bedaquiline formulation was stored at 4°C. The 160-mg/kg dose (based on an average mouse body mass of 20 g) was administered by two intramuscular injections (8 μl each, one into each hind thigh) using a BD Veo insulin syringe with a BD Ultra-Fine 3/10 ml 6-mm by 31-gauge needle with half-unit scale. Treatment was administered for up to 12 weeks, with five mice per treatment group being sacrificed at 4, 8, and 12 weeks after day 0. At sacrifice, the lungs were removed and homogenized, and CFU counts were determined as previously described (34) and as outlined in Tables S4 to S7; the dilution that yielded CFU counts closest to 50 was used to calculate the number of CFU per lung. At each time point, the bacterial burden of M. bovis rBCG30 was calculated using the values of the number of CFU per lung determined on hygromycin-containing agar. The M. tuberculosis implantation was calculated using the values of the number of CFU per lung determined on TCH-containing agar. The M. tuberculosis bacterial burdens at day 0 and weeks 4, 8, and 12 were calculated by subtracting the number of CFU per lung determined on hygromycin-containing agar from the total number of CFU per lung determined on plain agar. The primary outcome was the difference (decline) in M. tuberculosis lung CFU counts during treatment and the counts in the lungs of rBCG30-immunized but untreated negative-control mice.

### Statistical analyses.

CFU counts (*x*) were log transformed as (*x* + 1) before analysis. The bactericidal activity of different treatment regimens at each time point was compared using one-way analysis of variance with Bonferroni’s correction for multiple comparisons. Analyses were performed using GraphPad Prism (version 7.02) software.

## Supplementary Material

Supplemental file 1
