# Supplementary material for: Activity of a Long-Acting Injectable Bedaquiline Formulation in a Paucibacillary Mouse Model of Latent Tuberculosis Infection
Source: Antimicrob Agents Chemother. 2019 Mar 27;63(4):e00007-19. doi: 10.1128/AAC.00007-19 (PMC6437534; doi:10.1128/AAC.00007-19)
Supplement: Supplemental file 1 [file AAC.00007-19-s0001.pdf]

SUPPLEMENTAL MATERIALS

Figure S1. *M. tuberculosis* lung CFU counts during 12 weeks of treatment.
(Supplement Page 2)

Table S1. CFU count data from quantitative cultures of the bacterial suspensions used for aerosol infections.
(Supplement Page 3)

Table S2. CFU count data used to calculate the number of bacteria implanted in mouse lungs following aerosol infections.
(Supplement Page 4)

Table S3. CFU count data from quantitative cultures of mouse lung homogenates at Day 0.
(Supplement Page 5)

Table S4. CFU count data from quantitative cultures of mouse lung homogenates at Week 4.
(Supplement Page 6)

Table S5. CFU count data from quantitative cultures of mouse lung homogenates at Week 8.
(Supplement Page 8)

Table S6. CFU count data from quantitative cultures of mouse lung homogenates from untreated mice at Week 12.
(Supplement Page 11)

Table S7. CFU count data from quantitative cultures of mouse lung homogenates at Week 12.
(Supplement Page 12)

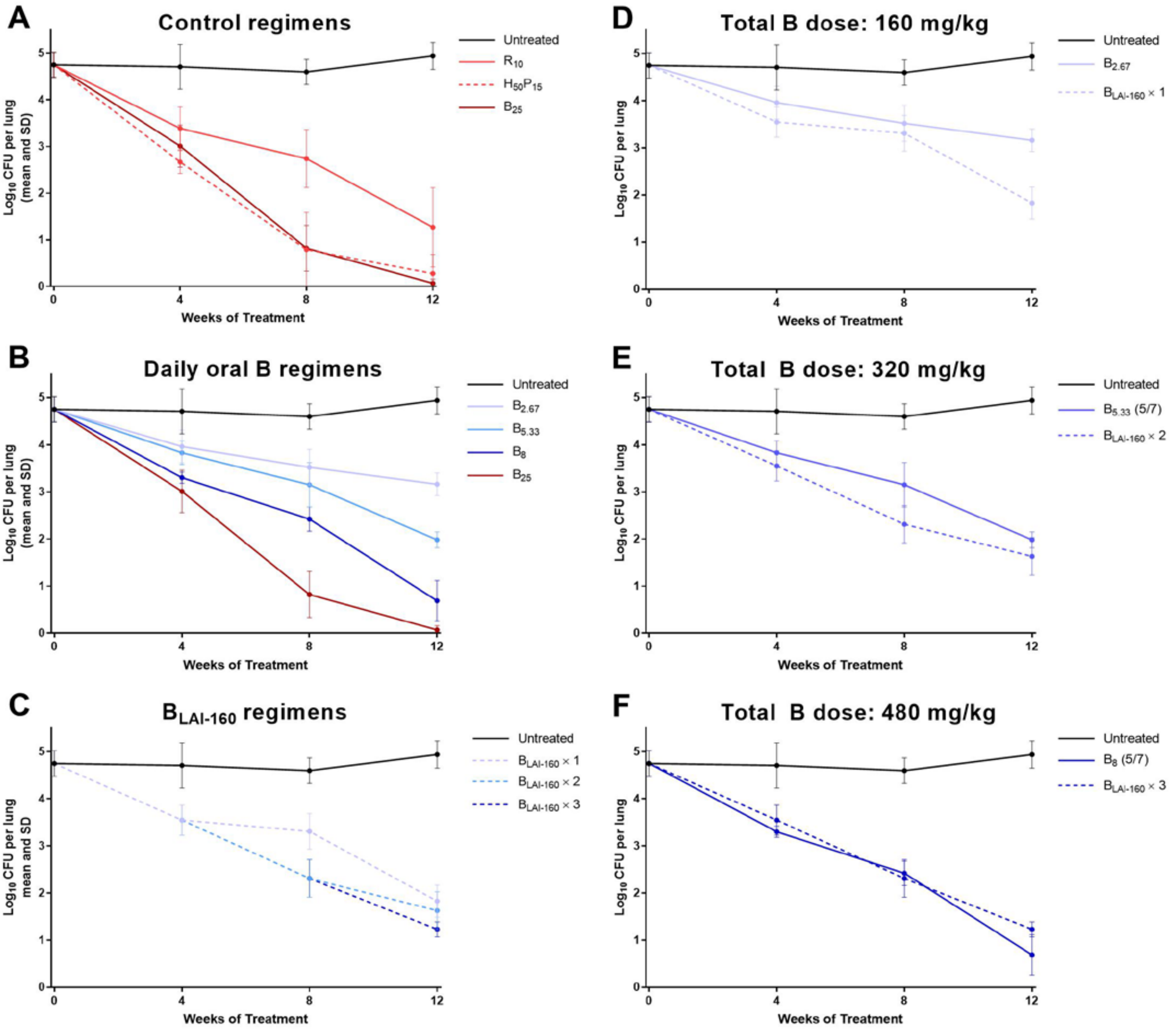

Figure S1. *M. tuberculosis* lung CFU counts during 12 weeks of treatment. Lung CFU data are presented for mice receiving (A) the control regimens, (B) the daily oral bedaquiline regimens, (C) the $B_{LAI-160}$ regimens, and total bedaquiline doses of (D) 160 mg/kg, (E) 320 mg/kg, and (F) 480 mg/kg. Data points represent mean values, and error bars represent standard deviation (n = 5 mice per group per time point) provided in **Table 3**. See **Table 2** for a description of the regimens. See **Tables S2-S7** for the raw CFU data at each time point.

Table S1. **CFU count data from quantitative cultures of the bacterial suspensions used for aerosol infections.** Serial ten-fold dilutions of the bacterial suspensions used for aerosol infections were plated on non-selective 7H11 agar (0.5 mL per agar plate). The shaded cells were used to calculate CFU/mL. The lower limits of detection were 4.30 $\log_{10}$ CFU/mL and 1.32 $\log_{10}$ CFU/mL for the *M. bovis* rBCG30 and *M. tuberculosis* H37Rv infections, respectively.

**Suspensions used for aerosol infections**	**CFU counts for the following 10-fold dilutions:**						**CFU/mL**	**$\log_{10}$ CFU/mL**
	**1**	**2**	**3**	**4**	**5**	**6**		
M. bovis rBCG30<br>OD_{600} = 1.03, adjusted to 0.56 and then diluted 5-fold	---	---	---	+	38	4	7 600 000	6.88
M. tuberculosis H37Rv<br>OD_{600} = 0.875, adjusted to 0.12 and then diluted 10-fold	++	+	27	3	---	---	54 000	4.73

++ indicates non-uniform confluent growth.
+ indicates individual colonies too numerous to accurately count or estimate.
--- indicates not determined.

Table S2. CFU count data used to calculate the number of bacteria implanted in mouse lungs following aerosol infections. Mice were sacrificed the day after infection. Undiluted lung homogenates and serial ten-fold dilutions were cultured (0.5 mL per agar plate) on selective 7H11 agar ("plain" agar) and selective 7H11 agar supplemented with 40 µg/mL HYG or 4 µg/mL TCH. The shaded cells were used to calculate CFU/lung for each sample.

Mouse number	Agar type	CFU counts for the following 10-fold dilutions:				CFU/lung	Log_{10} CFU/lung	LLOD
		0	1	2	3			
M. bovis **rBCG30 infection (Week -19)**								
1	Plain	40	14	1	---	700	2.85	0.78
	HYG	46	16	2	---	800	2.90	0.78
	TCH	---	0	---	---	0	0.00	1.71
2	Plain	11	18	2	---	900	2.95	0.78
	HYG	16	27	1	---	1 350	3.13	0.78
	TCH	---	0	---	---	0	0.00	1.71
3	Plain	12	23	0	---	1 150	3.06	0.78
	HYG	1	25	0	---	1 250	3.10	0.78
	TCH	---	0	---	---	0	0.00	1.71
4	Plain	40	26	0	---	1 300	3.11	0.78
	HYG	14	26	3	---	1 300	3.11	0.78
	TCH	---	0	---	---	0	0.00	1.71
5	Plain	7	24	3	---	1 200	3.08	0.78
	HYG	40	20	3	---	1 000	3.00	0.48
	TCH	---	0	---	---	0	0.00	1.71
M. tuberculosis **H37Rv infection (Week -13)**								
1	Plain	+	+	~150	21	105 000	5.02	0.78
	HYG	+	+	~150	17	85 000	4.93	0.78
	TCH	24	10	0	0	120	2.08	0.78
2	Plain	+	+	~150	12	60 000	4.78	0.78
	HYG	+	+	~150	15	75 000	4.88	0.78
	TCH	22	2	0	0	110	2.05	0.78
3	Plain	+	+	~200	19	95 000	4.98	0.78
	HYG	+	+	~200	28	140 000	5.15	0.78
	TCH	28	1	0	0	140	2.15	0.78
4	Plain	+	+	~150	10	50 000	4.70	0.78
	HYG	+	+	~150	17	85 000	4.93	0.78
	TCH	21	2	0	20	105	2.03	0.78
5	Plain	+	+	~150	17	85 000	4.93	0.78
	HYG	+	+	~150	15	75 000	4.88	0.78
	TCH	35	8	0	0	175	2.25	0.78

LLOD: lower limit of detection (log_{10} CFU/lung).
+ indicates individual colonies too numerous to accurately count or estimate.
~ indicates that a precise CFU count could not be determined due to merged/touching colonies.
--- indicates not determined.

Table S3. **CFU count data from quantitative cultures of mouse lung homogenates at Day 0.** Mouse lungs were homogenized in 2.5 mL PBS. Undiluted lung homogenate and serial ten-fold dilutions were cultured (0.5 mL per agar plate) on selective 7H11 agar ("plain" agar) and selective 7H11 agar supplemented with 40 µg/mL HYG or 4 µg/mL TCH. The shaded cells were used to calculate CFU/lung for each sample, and the lower limit of detection was 0.78 log_{10} CFU/lung for each sample.

Mouse number	Agar type	CFU counts for the following 10-fold dilutions:					CFU/lung	Log_{10} CFU/lung
		0	1	2	3	4		
1	Plain	++	+	57	2	0	28 500	4.45
	HYG	+	44	1	0	0	2 200	3.34
	TCH	++	+	66	1	0	33 000	4.52
2	Plain	++	+	146	9	1	45 000	4.65
	HYG	+	7	0	0	0	350	2.55
	TCH	++	+	188	13	2	65 000	4.81
3	Plain	++	++	212	16	2	80 000	4.90
	HYG	+	132	12	1	0	6 000	3.78
	TCH	++	++	256	19	0	95 000	4.98
4	Plain	++	+	116	10	0	50 000	4.70
	HYG	+	45	3	0	0	2 250	3.35
	TCH	++	+	122	11	0	55 000	4.74
5	Plain	++	++	223	28	4	140 000	5.15
	HYG	+	43	2	0	0	2 150	3.33
	TCH	++	++	214	28	2	140 000	5.15

++ indicates non-uniform confluent growth.
+ indicates individual colonies too numerous to accurately count or estimate.

Table S4. **CFU count data from quantitative cultures of mouse lung homogenates at Week 4.** Mouse lungs were homogenized in 3.0 mL PBS. Undiluted homogenates and up to three ten-fold serial dilutions of homogenates were plated (0.5 mL per agar plate) on selective 7H11 agar with charcoal ("Plain" agar) and selective 7H11 charcoal agar supplemented with 40 µg/mL HYG or 200 µg/mL TCH. The shaded cells were used to calculate CFU/lung for each sample.

LTBI treatment regimen	Mouse number	Agar type	CFU counts for the following 10-fold dilutions: 0	1	2	3	CFU/lung	Log_{10} CFU/lung	LLOD
Untreated	1	Plain	---	+	45	1	27 000	4.43	1.79
		HYG	---	50	3	0	3 000	3.48	1.79
		TCH	---	+	33	4	19 800	4.30	1.79
	2	Plain	---	+	92	10	60 000	4.78	1.79
		HYG	---	5	2	0	300	2.48	1.79
		TCH	---	+	66	8	39 600	4.60	1.79
	3	Plain	---	+	+	54	324 000	5.51	1.79
		HYG	---	112	13	1	7 800	3.89	1.79
		TCH	---	+	+	50	300 000	5.48	1.79
	4	Plain	---	+	33	1	19 800	4.30	1.79
		HYG	---	13	2	0	780	2.89	1.79
		TCH	---	+	26	1	15 600	4.19	1.79
	5	Plain	---	+	75	8	45 000	4.65	1.79
		HYG	---	71	8	3	4 260	3.63	1.79
		TCH	---	+	67	7	40 200	4.60	1.79
R_{10} (5/7)	1	Plain	68	11	1	---	660	2.82	0.85
		HYG	0	0	0	---	0	0.00	0.85
		TCH	56	7	0	---	336	2.53	0.85
	2	Plain	+	107	14	---	8 400	3.92	0.85
		HYG	0	0	0	---	0	0.00	0.85
		TCH	+	63	8	---	3 780	3.58	0.85
	3	Plain	Contam.	60	3	---	3 600	3.56	1.79
		HYG	0	0	0	---	0	0.00	0.85
		TCH	+	42	3	---	2 520	3.40	0.85
	4	Plain	+	17	1	---	1 020	3.01	0.85
		HYG	0	0	0	---	0	0.00	0.85
		TCH	+	16	3	---	960	2.98	0.85
	5	Plain	+	101	7	---	4 200	3.62	0.85
		HYG	1	0	0	---	6	0.85	0.85
		TCH	+	47	5	---	2 820	3.45	0.85
$H_{50}P_{15}$(1/7)	1	Plain	97	13	5	---	780	2.89	0.85
		HYG	1	0	0	---	6	0.85	0.85
		TCH	91	8	0	---	546	2.74	0.85
	2	Plain	89	15	0	---	900	2.95	0.85
		HYG	0	0	0	---	0	0.00	0.85
		TCH	77	10	1	---	462	2.67	0.85
	3	Plain	50	6	0	---	300	2.48	0.85
		HYG	0	0	0	---	0	0.00	0.85
		TCH	Contam.	3	0	---	180	2.26	1.79
	4	Plain	74	10	0	---	444	2.65	0.85
		HYG	0	0	0	---	0	0.00	0.85
		TCH	48	5	0	---	288	2.46	0.85
	5	Plain	40	1	0	---	240	2.38	0.85
		HYG	0	0	0	---	0	0.00	0.85
		TCH	29	2	0	---	174	2.24	0.85
B_{25} (5/7)	1	Plain	62	15	1	---	372	2.57	0.85
		HYG	0	0	0	---	0	0.00	0.85
		TCH	40	5	0	---	240	2.38	0.85
	2	Plain	53	6	2	---	318	2.50	0.85
		HYG	0	0	0	---	0	0.00	0.85
		TCH	28	5	0	---	168	2.23	0.85
	3	Plain	177	32	3	---	1 920	3.28	0.85
		HYG	0	0	0	---	0	0.00	0.85
		TCH	98	19	1	---	1 140	3.06	0.85
	4	Plain	+	54	8	---	3 240	3.51	0.85
		HYG	2	0	0	---	12	1.11	0.85
		TCH	+	38	2	---	2 280	3.36	0.85
	5	Plain	109	25	0	---	1 500	3.18	0.85
		HYG	1	0	0	---	6	0.85	0.85
		TCH	+	19	1	---	1 140	3.06	0.85

Continued on next page.

Table S4, continued.

LTBI treatment regimen	Mouse number	Agar type	CFU counts for the following 10-fold dilutions: 0	1	2	3	CFU/lung	Log_{10} CFU/lung	LLOD
B_8 (5/7)	1	Plain	+	35	1	0	2 100	3.32	0.85
		HYG	0	0	0	0	0	0.00	0.85
		TCH	+	18	0	0	1 080	3.03	0.85
	2	Plain	+	27	3	0	1 620	3.21	0.85
		HYG	0	0	0	0	0	0.00	0.85
		TCH	83	14	1	0	498	2.70	0.85
	3	Plain	120	26	0	0	1 560	3.19	0.85
		HYG	1	0	0	0	6	0.85	0.85
		TCH	88	6	0	0	528	2.72	0.85
	4	Plain	+	52	8	0	3 120	3.49	0.85
		HYG	0	0	0	0	0	0.00	0.85
		TCH	+	34	4	---	2 040	3.31	0.85
	5	Plain	+	33	0	0	1 980	3.30	0.85
		HYG	0	0	0	0	0	0.00	0.85
		TCH	+	13	2	0	780	2.89	0.85
$B_{5.33}$ (5/7)	1	Plain	+	65	5	1	3 900	3.59	0.85
		HYG	0	0	0	0	0	0.00	0.85
		TCH	+	29	3	0	1 740	3.24	0.85
	2	Plain	+	88	11	0	5 280	3.72	0.85
		HYG	3	0	0	0	18	1.28	0.85
		TCH	+	45	5	0	2 700	3.43	0.85
	3	Plain	+	165	28	2	16 800	4.23	0.85
		HYG	5	2	0	0	30	1.49	0.85
		TCH	+	72	7	3	4 320	3.64	0.85
	4	Plain	+	97	14	0	8 400	3.92	0.85
		HYG	1	0	0	0	6	0.85	0.85
		TCH	+	42	6	0	2 520	3.40	0.85
	5	Plain	+	79	9	0	4 740	3.68	0.85
		HYG	2	0	0	0	12	1.11	0.85
		TCH	+	31	4	0	1 860	3.27	0.85
$B_{2.67}$ (5/7)	1	Plain	+	71	5	0	4 260	3.63	0.85
		HYG	0	0	0	0	0	0.00	0.85
		TCH	Contam.	57	7	1	3 420	3.53	1.79
	2	Plain	+	+	54	1	32 400	4.51	0.85
		HYG	37	4	0	0	222	2.35	0.85
		TCH	+	+	30	1	18 000	4.26	0.85
	3	Plain	+	92	17	1	10 200	4.01	0.85
		HYG	12	3	0	0	72	1.86	0.85
		TCH	+	73	10	0	4 380	3.64	0.85
	4	Plain	+	77	12	0	4 620	3.66	0.85
		HYG	2	1	0	0	12	1.11	0.85
		TCH	+	30	7	0	1 800	3.26	0.85
	5	Plain	+	113	17	0	10 200	4.01	0.85
		HYG	7	0	0	0	42	1.63	0.85
		TCH	+	81	6	0	4 860	3.69	0.85
$B_{LAI-160}$ (1/28) × 1	1	Plain	+	124	15	1	9 000	3.95	0.85
		HYG	0	0	0	0	0	0.00	0.85
		TCH	+	71	5	0	4 260	3.63	0.85
	2	Plain	+	31	3	0	1 860	3.27	0.85
		HYG	0	0	0	0	0	0.00	0.85
		TCH	42	9	2	0	540	2.73	0.85
	3	Plain	+	74	6	0	4 440	3.65	0.85
		HYG	0	0	0	0	0	0.00	0.85
		TCH	+	44	3	0	2 640	3.42	0.85
	4	Plain	+	26	6	0	1 560	3.19	0.85
		HYG	2	0	0	0	12	1.11	0.85
		TCH	+	25	1	0	1 500	3.18	0.85
	5	Plain	+	81	8	2	4 860	3.69	0.85
		HYG	1	0	0	0	6	0.85	0.85
		TCH	+	37	5	1	2 220	3.35	0.85

LLOD: lower limit of detection (log_{10} CFU/lung).
Contam. indicates bacterial or fungal contamination; this plate was not used to calculate CFU/lung.
+ indicates individual colonies too numerous to accurately count or estimate.
--- indicates not determined.

Table S5. **CFU count data from quantitative cultures of mouse lung homogenates at Week 8.** Mouse lungs were homogenized in 3.0 mL PBS. Undiluted homogenates and up to three ten-fold serial dilutions of homogenates were plated (0.5 mL per agar plate) on selective 7H11 agar with charcoal ("Plain" agar) and selective 7H11 charcoal agar supplemented with 40 µg/mL HYG or 200 µg/mL TCH. The shaded cells were used to calculate CFU/lung for each sample.

LTBI treatment regimen	Mouse number	Agar type	CFU counts for the following 10-fold dilutions:				CFU/lung	Log_{10} CFU/lung	LLOD
			0	1	2	3			
Untreated	1	Plain	---	+	49	3	29 400	4.47	1.79
		HYG	---	58	4	0	3 480	3.54	1.79
		TCH	---	+	47	3	28 200	4.45	1.79
	2	Plain	---	+	77	6	46 200	4.66	1.79
		HYG	---	32	2	0	1 920	3.28	1.79
		TCH	---	+	79	4	47 400	4.68	1.79
	3	Plain	---	+	239	22	132 000	5.12	1.79
		HYG	---	+	30	4	18 000	4.26	1.79
		TCH	---	+	221	21	126 000	5.10	1.79
	4	Plain	---	+	49	4	29 400	4.47	1.79
		HYG	---	52	3	1	3 120	3.49	1.79
		TCH	---	+	50	1	30 000	4.48	1.79
	5	Plain	---	+	50	5	30 000	4.48	1.79
		HYG	---	10	0	0	600	2.78	1.79
		TCH	---	+	32	0	19 200	4.28	1.79
R_{10} (5/7)	1	Plain	123	14	0	---	840	2.92	0.85
		HYG	0	0	0	---	0	0.00	0.85
		TCH	128	7	1	---	420	2.62	0.85
	2	Plain	7	0	0	---	42	1.63	0.85
		HYG	0	0	0	---	0	0.00	0.85
		TCH	Contam.	0	0	---	---	---	1.79
	3	Plain	104	15	0	---	900	2.95	0.85
		HYG	0	0	0	---	0	0.00	0.85
		TCH	Contam.	Contam.	Contam.	---	---	---	---
	4	Plain	135	23	0	---	1 380	3.14	0.85
		HYG	0	0	0	---	0	0.00	0.85
		TCH	173	26	2	---	1 560	3.19	0.85
	5	Plain	113	18	2	---	1 080	3.03	0.85
		HYG	0	0	0	---	0	0.00	0.85
		TCH	112	10	0	---	600	2.78	0.85
$H_{50}P_{15}$(1/7)	1	Plain	11	0	0	---	66	1.83	0.85
		HYG	0	0	0	---	0	0.00	0.85
		TCH	Contam.	0	0	---	---	---	1.79
	2	Plain	3	0	0	---	18	1.28	0.85
		HYG	0	0	0	---	0	0.00	0.85
		TCH	Contam.	Contam.	0	---	---	---	2.78
	3	Plain	0	0	0	---	0	0.00	0.85
		HYG	0	0	0	---	0	0.00	0.85
		TCH	0	0	0	---	0	0.00	0.85
	4	Plain	0	0	0	---	0	0.00	0.85
		HYG	0	0	0	---	0	0.00	0.85
		TCH	0	0	0	---	0	0.00	0.85
	5	Plain	1	0	0	---	6	0.85	0.85
		HYG	0	0	0	---	0	0.00	0.85
		TCH	0	0	0	---	0	0.00	0.85

Continued on next page.

Table S5, continued.

LTBI treatment regimen	Mouse number	Agar type	CFU counts for the following 10-fold dilutions: 0	1	2	3	CFU/lung	Log_{10} CFU/lung	LLOD
B_{25} (5/7)	1	Plain	3	0	0	---	18	1.28	0.85
		HYG	0	0	0	---	0	0.00	0.85
		TCH	4	0	0	---	24	1.40	0.85
	2	Plain	1	0	0	---	6	0.85	0.85
		HYG	0	0	0	---	0	0.00	0.85
		TCH	3	0	0	---	18	1.28	0.85
	3	Plain	1	0	0	---	6	0.85	0.85
		HYG	0	0	0	---	0	0.00	0.85
		TCH	0	0	0	---	0	0.00	0.85
	4	Plain	2	1	0	---	12	1.11	0.85
		HYG	0	0	0	---	0	0.00	0.85
		TCH	0	0	0	---	0	0.00	0.85
	5	Plain	0	0	0	---	0	0.00	0.85
		HYG	0	0	0	---	0	0.00	0.85
		TCH	0	0	0	---	0	0.00	0.85
B_8 (5/7)	1	Plain	16	1	0	0	96	1.99	0.85
		HYG	0	0	0	0	0	0.00	0.85
		TCH	10	1	0	0	60	1.79	0.85
	2	Plain	73	16	1	0	438	2.64	0.85
		HYG	0	0	0	0	0	0.00	0.85
		TCH	69	6	0	0	414	2.62	0.85
	3	Plain	61	4	4	0	366	2.56	0.85
		HYG	0	0	0	0	0	0.00	0.85
		TCH	37	6	0	0	222	2.35	0.85
	4	Plain	53	8	0	0	318	2.50	0.85
		HYG	0	0	0	0	0	0.00	0.85
		TCH	32	3	0	0	192	2.29	0.85
	5	Plain	42	7	1	0	252	2.40	0.85
		HYG	0	0	0	0	0	0.00	0.85
		TCH	31	1	0	0	186	2.27	0.85
$B_{5.33}$ (5/7)	1	Plain	109	13	0	0	780	2.89	0.85
		HYG	0	0	0	0	0	0.00	0.85
		TCH	48	9	0	0	288	2.46	0.85
	2	Plain	+	65	8	2	3 900	3.59	0.85
		HYG	0	0	0	0	0	0.00	0.85
		TCH	Contam.	40	1	0	2 400	3.38	1.79
	3	Plain	+	77	9	0	4 620	3.66	0.85
		HYG	0	0	0	0	0	0.00	0.85
		TCH	98	19	2	0	1 140	3.06	0.85
	4	Plain	64	12	0	0	384	2.59	0.85
		HYG	0	0	0	0	0	0.00	0.85
		TCH	29	3	0	0	174	2.24	0.85
	5	Plain	101	17	2	0	1 020	3.01	0.85
		HYG	1	0	0	0	6	0.85	0.85
		TCH	60	11	2	0	360	2.56	0.85

Continued on next page.

Table S5, continued.

LTBI treatment regimen	Mouse number	Agar type	CFU counts for the following 10-fold dilutions: 0	1	2	3	CFU/lung	Log_{10} CFU/lung	LLOD
$B_{2.67}$ (5/7)	1	Plain	+	84	8	0	5 040	3.70	0.85
		HYG	6	0	0	0	36	1.57	0.85
		TCH	+	56	2	0	3 360	3.53	0.85
	2	Plain	+	126	15	1	9 000	3.95	0.85
		HYG	14	2	0	0	84	1.93	0.85
		TCH	+	81	10	0	4 860	3.69	0.85
	3	Plain	+	87	9	0	5 220	3.72	0.85
		HYG	1	0	0	0	6	0.85	0.85
		TCH	+	33	3	0	1 980	3.30	0.85
	4	Plain	+	18	4	0	1 080	3.03	0.85
		HYG	4	0	0	0	24	1.40	0.85
		TCH	61	20	3	0	366	2.56	0.85
	5	Plain	+	28	1	1	1 680	3.23	0.85
		HYG	0	0	0	0	0	0.00	0.85
		TCH	103	11	0	0	660	2.82	0.85
$B_{LAI\text{-}160}$ (1/28) × 2	1	Plain	13	3	0	0	78	1.90	0.85
		HYG	0	0	0	0	0	0.00	0.85
		TCH	8	0	0	0	48	1.69	0.85
	2	Plain	34	3	1	0	204	2.31	0.85
		HYG	0	0	0	0	0	0.00	0.85
		TCH	19	1	0	0	114	2.06	0.85
	3	Plain	16	1	0	0	96	1.99	0.85
		HYG	0	0	0	0	0	0.00	0.85
		TCH	8	0	0	0	48	1.69	0.85
	4	Plain	50	7	1	0	300	2.48	0.85
		HYG	0	0	0	0	0	0.00	0.85
		TCH	31	2	1	0	186	2.27	0.85
	5	Plain	Contam.	13	0	0	780	2.89	1.79
		HYG	0	0	0	0	0	0.00	0.85
		TCH	Contam.	21	1	1	1 260	3.10	1.79
$B_{LAI\text{-}160}$ (1/28) × 1	1	Plain	99	7	1	0	594	2.77	0.85
		HYG	1	0	0	0	6	0.85	0.85
		TCH	65	13	0	0	390	2.59	0.85
	2	Plain	+	48	7	0	2 880	3.46	0.85
		HYG	0	0	0	0	0	0.00	0.85
		TCH	+	35	5	0	2 100	3.32	0.85
	3	Plain	+	81	7	1	4 860	3.69	0.85
		HYG	12	2	0	0	72	1.86	0.85
		TCH	+	69	7	0	4 140	3.62	0.85
	4	Plain	109	20	1	0	1 200	3.08	0.85
		HYG	0	0	0	0	0	0.00	0.85
		TCH	93	19	1	0	1 140	3.06	0.85
	5	Plain	+	63	4	0	3 780	3.58	0.85
		HYG	0	0	0	0	0	0.00	0.85
		TCH	+	22	3	0	1 320	3.12	0.85

LLOD: lower limit of detection (log_{10} CFU/lung).
Contam. indicates bacterial or fungal contamination; this plate was not used to calculate CFU/lung.
+ indicates individual colonies too numerous to accurately count or estimate.
--- indicates not determined.

Table S6. **CFU count data from quantitative cultures of mouse lung homogenates from untreated mice at Week 12.** Mouse lungs were homogenized in 3.0 mL PBS. Four ten-fold serial dilutions of lung homogenates were plated (0.5 mL per agar plate) on selective 7H11 agar with charcoal ("Plain" agar) and selective 7H11 charcoal agar supplemented with 40 µg/mL HYG or 200 µg/mL TCH. The shaded cells were used to calculate CFU/lung for each sample.

Mouse number	Agar type	CFU counts for the following 10-fold dilutions:				CFU/lung	Log_{10} CFU/lung	LLOD
		1	2	3	4			
1	Plain	+	190	27	2	162 000	5.21	1.79
	HYG	17	3	0	0	1 020	3.01	1.79
	TCH	+	187	25	1	150 000	5.18	1.79
2	Plain	+	130	11	1	66 000	4.82	1.79
	HYG	57	8	1	0	3 420	3.53	1.79
	TCH	+	137	9	4	54 000	4.73	1.79
3	Plain	Contam.	Contam.	10	1	60 000	4.78	3.78
	HYG	27	3	1	0	1 620	3.21	1.79
	TCH	+	97	7	1	42 000	4.62	1.79
4	Plain	++	300	32	3	192 000	5.28	1.79
	HYG	3	0	0	0	180	2.26	1.79
	TCH	++	+	31	2	186 000	5.27	1.79
5	Plain	+	76	7	0	45 600	4.66	1.79
	HYG	37	3	0	0	2 220	3.35	1.79
	TCH	+	53	4	0	31 800	4.50	1.79

LLOD: lower limit of detection (log_{10} CFU/lung).
Contam. indicates bacterial or fungal contamination; this plate was not used to calculate CFU/lung.
++ indicates non-uniform confluent growth.
+ indicates individual colonies too numerous to accurately count or estimate.

Table S7. **CFU count data from quantitative cultures of mouse lung homogenates at Week 12.** Mouse lungs were homogenized in 3.0 mL PBS. Undiluted homogenates and up to two ten-fold serial dilutions of homogenates were plated (0.5 mL per agar plate) on selective 7H11 agar with charcoal (“Plain” agar) and selective 7H11 charcoal agar supplemented with 40 µg/mL HYG or 200 µg/mL TCH. The shaded cells were used to calculate CFU/lung for each sample.

LTBI treatment regimen	Mouse number	Agar type	CFU counts for the following 10-fold dilutions:						CFU/lung	Log_{10} CFU/lung	LLOD
			Undiluted				1	2			
			Plate 1	Plate 2	Plate 3	Plate 4					
R_{10} (5/7)	1	Plain	0	1	1	0	0	---	4	0.68	0.40
		HYG	0	---	---	---	0	---	0	0.00	0.85
		TCH	---	---	---	---	---	---	---	---	---
	2	Plain	0	2	1	0	0	---	6	0.82	0.40
		HYG	0	---	---	---	0	---	0	0.00	0.85
		TCH	---	---	---	---	---	---	---	---	---
	3	Plain	78	69	81	65	10	---	549	2.74	0.40
		HYG	0	---	---	---	0	---	0	0.00	0.85
		TCH	---	---	---	---	---	---	---	---	---
	4	Plain	2	2	5	1	0	---	19	1.30	0.40
		HYG	0	---	---	---	0	---	0	0.00	0.85
		TCH	---	---	---	---	---	---	---	---	---
	5	Plain	0	1	1	1	0	---	6	0.82	0.40
		HYG	0	---	---	---	0	---	0	0.00	0.85
		TCH	---	---	---	---	---	---	---	---	---
$H_{50}P_{15}$(1/7)	1	Plain	0	0	Contam.	Contam.	0	---	0	0.00	0.60
		HYG	0	---	---	---	0	---	0	0.00	0.85
		TCH	---	---	---	---	---	---	---	---	---
	2	Plain	0	0	0	0	0	---	0	0.00	0.40
		HYG	0	---	---	---	0	---	0	0.00	0.85
		TCH	---	---	---	---	---	---	---	---	---
	3	Plain	1	0	0	0	0	---	2	0.46	0.40
		HYG	0	---	---	---	0	---	0	0.00	0.85
		TCH	---	---	---	---	---	---	---	---	---
	4	Plain	0	0	0	0	0	---	0	0.00	0.40
		HYG	0	---	---	---	0	---	0	0.00	0.85
		TCH	---	---	---	---	---	---	---	---	---
	5	Plain	1	1	1	1	1	---	8	0.93	0.40
		HYG	0	---	---	---	0	---	0	0.00	0.85
		TCH	---	---	---	---	---	---	---	---	---
B_{25} (5/7)	1	Plain	Contam.	0	0	0	---	---	0	0.00	0.48
		HYG	0	---	---	---	---	---	0	0.00	0.85
		TCH	---	---	---	---	---	---	---	---	---
	2	Plain	0	0	1	0	---	---	2	0.46	0.40
		HYG	0	---	---	---	---	---	0	0.00	0.85
		TCH	---	---	---	---	---	---	---	---	---
	3	Plain	1	0	0	0	---	---	2	0.46	0.40
		HYG	0	---	---	---	---	---	0	0.00	0.85
		TCH	---	---	---	---	---	---	---	---	---
	4	Plain	0	0	0	0	---	---	0	0.00	0.40
		HYG	0	---	---	---	---	---	0	0.00	0.85
		TCH	---	---	---	---	---	---	---	---	---
	5	Plain	0	0	0	0	---	---	0	0.00	0.40
		HYG	0	---	---	---	---	---	0	0.00	0.85
		TCH	---	---	---	---	---	---	---	---	---
B_8 (5/7)	1	Plain	2	0	1	Contam.	0	0	8	0.93	0.48
		HYG	0	---	---	---	---	---	0	0.00	0.85
		TCH	---	---	---	---	---	---	---	---	---
	2	Plain	1	0	1	0	0	0	4	0.68	0.40
		HYG	0	---	---	---	---	---	0	0.00	0.85
		TCH	---	---	---	---	---	---	---	---	---
	3	Plain	0	0	0	2	0	0	4	0.68	0.40
		HYG	0	---	---	---	---	---	0	0.00	0.85
		TCH	---	---	---	---	---	---	---	---	---
	4	Plain	0	0	0	0	---	---	0	0.00	0.40
		HYG	0	---	---	---	---	---	0	0.00	0.85
		TCH	---	---	---	---	---	---	---	---	---
	5	Plain	0	0	4	3	0	0	13	1.15	0.40
		HYG	0	---	---	---	---	---	0	0.00	0.85
		TCH	---	---	---	---	---	---	---	---	---

Continued on next page.

Table S7, continued.

LTBI treatment regimen	Mouse number	Agar type	CFU counts for the following 10-fold dilutions:						CFU/lung	Log_{10} CFU/lung	LLOD
			Undiluted				1	2			
			Plate 1	Plate 2	Plate 3	Plate 4					
$B_{5.33}$ (5/7)	1	Plain	28	14	23	25	1	---	169	2.23	0.40
		HYG	3	---	---	---	0	0	23	1.37	0.85
		TCH	13	---	---	---	1	0	98	1.99	0.85
	2	Plain	10	8	7	11	---	---	68	1.84	0.40
		HYG	1	---	---	---	0	---	8	0.93	0.85
		TCH	2	---	---	---	1	---	15	1.20	0.85
	3	Plain	29	17	16	14	2	0	143	2.16	0.40
		HYG	0	---	---	---	0	0	0	0.00	0.85
		TCH	14	---	---	---	2	0	105	2.03	0.85
	4	Plain	12	8	11	Contam.	---	---	78	1.89	0.48
		HYG	1	---	---	---	0	---	8	0.93	0.85
		TCH	10	---	---	---	1	0	75	1.88	0.85
	5	Plain	15	13	12	7	1	0	88	1.95	0.40
		HYG	0	---	---	---	0	---	0	0.00	0.85
		TCH	14	---	---	---	1	0	105	2.03	0.85
$B_{2.67}$ (5/7)	1	Plain	227	218	---	---	43	1	2 580	3.41	0.60
		HYG	23	---	---	---	8	2	173	2.24	0.85
		TCH	+	---	---	---	30	2	1 800	3.26	0.85
	2	Plain	190	107	---	---	27	2	1 620	3.21	0.60
		HYG	52	---	---	---	5	0	390	2.59	0.85
		TCH	+	---	---	---	28	3	1 680	3.23	0.85
	3	Plain	235	212	---	---	29	5	1 740	3.24	0.60
		HYG	60	---	---	---	9	0	450	2.65	0.85
		TCH	+	---	---	---	44	5	2 640	3.42	0.85
	4	Plain	201	207	---	---	48	4	2 880	3.46	0.60
		HYG	44	---	---	---	6	0	330	2.52	0.85
		TCH	+	---	---	---	33	2	1 980	3.30	0.85
	5	Plain	312	397	---	---	62	5	3 720	3.57	0.60
		HYG	52	---	---	---	11	0	390	2.59	0.85
		TCH	+	---	---	---	51	3	3 060	3.49	0.85
$B_{LAI-160}$ (1/28) × 3	1	Plain	1	4	3	4	0	0	23	1.37	0.40
		HYG	1	---	---	---	0	0	8	0.93	0.85
		TCH	---	---	---	---	---	---	---	---	---
	2	Plain	3	2	2	1	0	0	15	1.20	0.40
		HYG	0	---	---	---	0	0	0	0.00	0.85
		TCH	---	---	---	---	---	---	---	---	---
	3	Plain	1	2	1	2	0	0	11	1.09	0.40
		HYG	0	---	---	---	0	0	0	0.00	0.85
		TCH	---	---	---	---	---	---	---	---	---
	4	Plain	2	4	0	1	0	0	13	1.15	0.40
		HYG	0	---	---	---	0	0	0	0.00	0.85
		TCH	---	---	---	---	---	---	---	---	---
	5	Plain	4	6	3	3	1	0	30	1.49	0.40
		HYG	0	---	---	---	0	0	0	0.00	0.85
		TCH	---	---	---	---	---	---	---	---	---
$B_{LAI-160}$ (1/28) × 2	1	Plain	16	20	19	23	3	0	146	2.17	0.40
		HYG	2	---	---	---	0	0	15	1.20	0.85
		TCH	15	---	---	---	2	0	113	2.05	0.85
	2	Plain	5	2	4	3	0	0	26	1.44	0.40
		HYG	2	---	---	---	0	0	15	1.20	0.85
		TCH	2	---	---	---	0	0	15	1.20	0.85
	3	Plain	10	3	8	9	0	0	56	1.76	0.40
		HYG	1	---	---	---	0	0	8	0.93	0.85
		TCH	8	---	---	---	1	0	60	1.79	0.85
	4	Plain	4	5	3	1	0	0	24	1.40	0.40
		HYG	0	---	---	---	0	0	0	0.00	0.85
		TCH	5	---	---	---	1	0	38	1.59	0.85
	5	Plain	11	7	7	10	3	---	66	1.82	0.40
		HYG	0	---	---	---	0	0	0	0.00	0.85
		TCH	6	---	---	---	0	0	45	1.66	0.85
$B_{LAI-160}$ (1/28) × 1	1	Plain	17	14	9	17	2	0	107	2.03	0.40
		HYG	3	---	---	---	0	0	23	1.37	0.85
		TCH	23	---	---	---	2	1	173	2.24	0.85
	2	Plain	8	6	5	12	0	0	58	1.77	0.40
		HYG	1	---	---	---	0	0	8	0.93	0.85
		TCH	5	---	---	---	0	0	38	1.59	0.85
	3	Plain	35	55	78	Contam.	12	0	420	2.62	0.48
		HYG	26	---	---	---	6	0	195	2.29	0.85
		TCH	60	---	---	---	11	1	450	2.65	0.85
	4	Plain	8	8	4	6	1	0	49	1.70	0.40
		HYG	3	---	---	---	0	0	23	1.37	0.85
		TCH	8	---	---	---	1	0	60	1.79	0.85
	5	Plain	6	9	7	6	0	---	53	1.73	0.40
		HYG	0	---	---	---	0	0	0	0.00	0.85
		TCH	3	---	---	---	1	0	23	1.37	0.85

LLOD: lower limit of detection (log_{10} CFU/lung). Contam. indicates bacterial or fungal contamination; this plate was not used to calculate CFU/lung. ++ indicates non-uniform confluent growth. + indicates individual colonies too numerous to accurately count or estimate.
